# Origin, Behaviour, and Transmission of B Chromosome with Special Reference to *Plantago lagopus*

**DOI:** 10.3390/genes10020152

**Published:** 2019-02-18

**Authors:** Manoj K. Dhar, Jasmeet Kour, Sanjana Kaul

**Affiliations:** Genome Research Laboratory, School of Biotechnology, University of Jammu, Jammu-180006, India; jassi29honey@gmail.com (J.K.); sanrozie@rediffmail.com (S.K.)

**Keywords:** B chromosome, transmission, origin, drive

## Abstract

B chromosomes have been reported in many eukaryotic organisms. These chromosomes occur in addition to the standard complement of a species. Bs do not pair with any of the A chromosomes and they have generally been considered to be non-essential and genetically inert. However, due to tremendous advancements in the technologies, the molecular composition of B chromosomes has been determined. The sequencing data has revealed that B chromosomes have originated from A chromosomes and they are rich in repetitive elements. In our laboratory, a novel B chromosome was discovered in *Plantago lagopus*. Using molecular cytogenetic techniques, the B chromosome was found to be composed of ribosomal DNA sequences. However, further characterization of the chromosome using next generation sequencing (NGS) etc. revealed that the B chromosome is a mosaic of sequences derived from A chromosomes, 5S ribosomal DNA (rDNA), 45S rDNA, and various types of repetitive elements. The transmission of B chromosome through the female sex track did not follow the Mendelian principles. The chromosome was found to have drive due to which it was perpetuating in populations. The present paper attempts to summarize the information on nature, transmission, and origin of B chromosomes, particularly the current status of our knowledge in *P. lagopus*.

## 1. Introduction

B chromosomes are enigmatic elements of the genome of some organisms. Interestingly, these chromosomes do not recombine with A chromosomes and have thus followed a different pathway during evolution [1]. These are also called as non-essential, extra, or supernumerary chromosomes because of their inertness. B chromosomes have been reported in over 2000 plant, animal, and fungal species [2]. The distribution of B chromosomes within an individual or among different individuals of a population is not uniform; they are not found in all members of a population, and their copy number can also vary among individuals possessing Bs. Since the B chromosomes remain unpaired during meiosis, as a result their mode of inheritance is irregular. Due to their dispensable nature, Bs are considered non-functional or parasitic elements, since they use the cellular machinery that is required for the maintenance and inheritance of A chromosomes [2]. Although B chromosomes are not essential, some phenotypic effects have been reported, which are usually cumulative depending upon the number and not the presence or absence of Bs. When present in low numbers, Bs have little, if any, influence on the phenotype, but at higher numbers they mostly have a negative effect on the fitness and fertility of the organism [3]. It was found that Maize B chromosomes influence the A-genome transcription with stronger effect associated with an increase in the copy number of B chromosome. Huang et al., compared lines with and without the B chromosome and detected 130 differentially expressed genes [4]. Despite being deleterious to the host genome at high copy numbers, Bs survive in numerous populations across multiple eukaryotic kingdoms. There is evidence that Bs directly or indirectly influence the behavior of A chromosomes [1]. These chromosomes acquire transposons, repetitive DNA, and organellar DNA. Bs have also been shown to harbor transcribed genes [4] and noncoding loci [5]. B chromosomes have been reported to contain thousands of sequences duplicated from essentially every chromosome in the ancestral karyotype [6].

Bs are widely distributed among eukaryotes: about 15% of eukaryotes contain these chromosomes [6]. There is considerable heterogeneity between different groups and also hotspots of occurrence and their presence is correlated with genome size [7]. Though there have been a number of reports on B chromosomes but a comprehensive theory for the origin, composition, regulation, maintenance, and evolution of B chromosomes has not emerged.

Recent technological advances in sequencing and genome analysis [8,9] have significantly enhanced our insight into the biology of B chromosomes. The intent of this review is to summarize the recent findings on B chromosomes with a pre-eminent focus on the B chromosome of *Plantago lagopus*.

*Plantago* L. is a large genus of annual or perennial herbs and subshrubs with a wide distribution. It is the chemical properties and divergent breeding systems (gynodioecy, self-incompatibility, and male sterility), due to which *Plantago* has grabbed the attention of many researchers [10]. *Plantago lagopus* L. is a small (about 30 cm tall), annual herb. It grows as a weed in the Mediterranean region. The diploid chromosome number of the species is 2n = 2x = 12. A spontaneous trisomic plant (2n = 2x = 13, Triplo 4) of *P. lagopus* was recovered in 1982 in an experimental population [11], which was a product of a nondisjunction event. When these trisomic plants were crossed with euploids, a remarkable plasticity of the genome was revealed, and plants having 1 to 16 chromosomes in addition to the standard set of 12 chromosomes, were recovered [12]. In one of the aneuploids, a primary trisomic for chromosome 2, the extra chromosome underwent rapid changes, resulting in the formation of ring chromosomes and chromosome fragments. Finally, the extra chromosome got stabilized as the proto-supernumerary isochromosome. Dhar et al., reported a novel B chromosome in *P. lagopus*, whose main body is composed of 5S ribosomal DNA (rDNA) and has few 45S rDNA sequences at the ends [12]. Here, we would review the work that aided in the identification and characterization of B chromosome in *P. lagopus*, which in turn helped in understanding the origin and evolution of B chromosomes.

## 2. Distribution of B Chromosomes

B chromosomes are widely distributed among eukaryotes [13], however, due to difficulty in preparing good cytological preparations in some taxa, possibly many species possessing Bs remain unknown at present. It has been estimated that Bs occur in 15% of eukaryotic species [2] and most of these are plants [14]. As far as animals are concerned, Bs are completely absent in birds [15] and are limited in mammals, where B chromosomes have been reported in 75 species out of about 5000 known species, which have been karyotyped [16]. There are a number of factors that have been related with the distribution of Bs among various species, namely: breeding system, genome size, and also the A chromosome morphology in certain mammals [7,17]. In genus *Plantago*, B chromosomes have been reported in *P. lagopus* [12], *Plantago major*, *Plantago lanceolata*, and tetraploid cytotypes of *Plantago depressa* [18], *Plantago seraria* [19], *Plantago boissieri* [20], and *Plantago coronopus* [21].

The distribution of Bs among major groups of angiosperm lineages and among lineages within families is nonrandom [13], but widespread. While B chromosomes are present in only 3% of eudicots, 8% of studied monocots among angiosperms possess B chromosomes [17], with considerable heterogeneity between different groups in frequency at the orders, families, and generic level. There are also hot spots of occurrence in Liliales and Commelinales [13]. Within the monocots, there are apparent differences between different families in B frequency. Some monocot families, such as the Melanthiaceae, have Bs in over 40% of the studied species, while others lack Bs entirely [17].

Frequency of Bs is independent of genome ploidy, with virtually no difference in frequency between diploids and polyploids [7,22]. Bs have been found to be more common in families with larger genomes, are found more frequently in mammals with acrocentric chromosomes [7,17,22]. It was hypothesized that there is less probability of presence of B chromosome in species with small genomes as larger genomes would better tolerate additional genetic material [17]. The presence of Bs has been positively correlated with pollution and/or stressful climactic environments in vertebrates [2].

Bs are carried by all the cells within the individual. However, there are some exceptions in which the Bs show lot of instability during mitosis in somatic tissues and therefore, they are present or absent in variable numbers in specific tissues and/or organs. In plants, only one-third of the investigated species display constancy of Bs in different tissues of the plant [23]. Absence of Bs from roots has been observed in *Erianthus munja* and *Erianthus ravennae* [13]. In few species the instability of the Bs is partial and strictly defined, e.g., in *Sorghum stipoideum*, mosaicism of B chromosomes has been reported in tapetal cells and microsporocytes, while they were totally eliminated from the leaves and stem [13]. In has been observed in some animals that the presence of B chromosomes leads to irregularities. For example, in *Locusta migratoria*, inter- and intrafollicular numerical variation has been reported [24]. The number of Bs varies between the cells of the testis in *Acris crepitans* [25]. Bs are generally stable among monocots; in *Allium* Bs are mostly unstable [26]. In *Brachycome dichromosomatica*, the large B has been found to be mitotically stable and the micro B varies in number from cell to cell [27].

In *P. lagopus*, the morphology of plants with and without B chromosome was found to be highly similar, indicating that the presence or absence of the B chromosome does not affect plant growth habit or vigour [12]. The distribution of B chromosome in this genus can also be associated with genome size, as this wild species *P. lagopus* with larger genome size (1.25 Gb) possesses B chromosome as compared to the other known species with smaller genome size, like *Plantago ovata* (0.65 Gb).

## 3. Structure of B Chromosomes

It is expected of B chromosome to show frequent polymorphisms because of its gratuitous nature. Undeniably, apart from a number of numeric polymorphisms, considerable structural polymorphisms of B chromosomes have been reported in a number of plants, e.g., *Aegilops speltoides* [28] and *B. dichromosomatica* [27]. Nevertheless, in some species like rye, B chromosomes have shown an identical cytological and molecular structure at the level of subspecies [1].

B chromosomes show variation in size also. The morphology of B chromosomes differs from that of A and are generally heterochromatinized. In plants, these chromosomes are often smaller than the smallest A, nevertheless mammalian Bs have been found within the size range of the As [2]. There are examples where B chromosomes larger than A chromosomes have been reported e.g., in the grasshopper *Eyprepocnemis plorans* [1], the cyprinid fish *Alburnus alburnus* [29], and the neotropical fish *Astyanax paranae* [30], though they are generally smaller than A chromosomes. In some species even, different types of B chromosomes coexist, e.g., in *B. dichromosomatica* [31] and the marsupial frog *Gastrotheca espeletia* [32].

## 4. Molecular Composition

Though B chromosomes have been largely studied, however, information about their molecular composition is scanty. In view of the possibility of origin of Bs from As, it was expected that the B chromosome would share sequence and structural similarity with A chromosome, so that they could synapse and recombine. However, this initial similarity seems to have been lost due to the accumulation of structural changes. Though, initially, experiments like density gradient centrifugation and renaturation kinetics showed similarity between A and B chromosome DNA composition, in recent years techniques like chromosome microdissection [32] and flow sorting [33] helped in the direct isolation of B chromosome-derived DNA. Next generation sequencing (NGS) has played a major role in the characterization of B chromosomes in some species. B chromosomes in rye have been characterized by using flow sorted A and B chromosomes [34]. Next generation sequencing has also been used to study the evolution and origin of B chromosome in the Cichlid fish *Astatotilapia latifasciata* [6] and *P. lagopus* [35]. These techniques showed how the composition of B chromosomes differs from A chromosomes, which was expected as the B chromosomes follow a separate evolutionary path.

One of the many exceptional features of B chromosomes is the accumulation of repetitive DNAs. Most of the B chromosomes reported in different organisms are heterochromatic mainly due to the presence of repetitive DNA sequences made of satellite DNA, ribosomal DNA, and transposable elements [34]. The type and copy number of repeats vary in B chromosomes. In many cases, it has been observed that B chromosomes contain much larger amounts of repetitive DNA when compared to the genome from which they originated, thus suggesting the massive amplification of repeat motifs over a relatively short time-scale [12].

B chromosomes have been reported to be enriched in repetitive sequences e.g., the micro B chromosome of *B. dichromosomatica*, which is mainly composed of tandem repeats [32], mobile elements, or non-coding repetitive sequences in *Crepis capillaris*, *Nectria haematococca*, and *Drosophila* [1]. Highly repetitive sequences, including transposons, were found to be present on both A and B chromosomes, but enriched on Bs in maize [36,37]. In case of rye, B chromosomes were found to accumulate large amounts of specific repeats and insertions of organellar DNA [34]. According to Valente et al., in Cichlid fish (*A. latifasciata*), the B chromosome originated early in the evolutionary history from a small fragment of one autosome [6]. While studying the B-linked genes of *Drosophila albomicans*, it was observed that 5.5% of the genome consisted of repetitive elements as compared to 5.35% of *Drosophila melanogaster* [38]. Since B chromosomes do not take part in recombination, they acquire larger fraction of mobile elements as compared to A chromosomes, which also leads to their heterochromatization. B chromosomes because of their relaxed selective pressure provide a safe spot for mobile elements [39].

**Figure 1:**
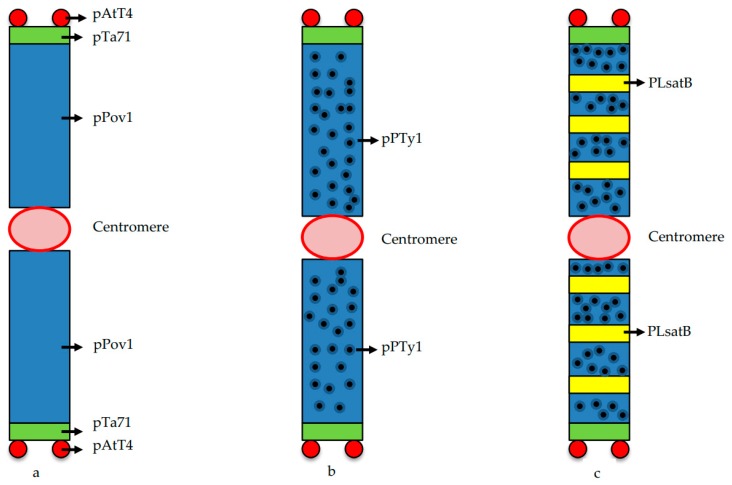
Deducing the composition of B chromosome in *Plantago lagopus* using FISH. (**a**) FISH signals reported on B chromosome using probes pTa71 (45S rDNA), pAtT4 (Telomere) and pPov1 (5S rDNA) [12]. Note painting of B chromosome by pPov1. (**b**) FISH signals for Ty-1 Copia elements reported over the entire length of B chromosome using probe pPTy1 [10]. (**c**) Specific localization of B specific satellite sequence, PLsatB using FISH on B chromosome [35]. FISH: Fluorescence in situ hybridization; rDNA: Ribosomal DNA.

The B chromosome in *P. lagopus* is cytogenetically unique. The chromosome was found to be completely heterochromatic using C-banding analysis [12]. The structure and behavior of B chromosomes was found to be typical of many naturally occurring B chromosomes, but it was unique in having its entire body mass derived from 5S DNA sequences [12]. The authors presented the experimental evidence of de novo origin of novel B chromosome in *P. lagopus* through specific DNA sequence amplification. Southern blot analysis revealed a distinct band of about 23 kb, only in +B plants. Using fluorescence in situ hybridization (FISH) and reverse genomic in situ hybridization (GISH) techniques, the entire chromosome was found to get painted with 5S rDNA probe, while 45S rDNA sequences were localized at the two ends, just below the telomeric sequences. Using molecular cytogenetic techniques, like FISH and Fiber-FISH, Kour et al., further characterized this chromosome and reported it to be a mixture of rDNA sequences and transposable elements [10]. It was also concluded that while 45S rDNA sequences are restricted to the subtelomeric regions, the 5S rDNA sequences are interspersed with transposons in the body of the B chromosome. Recently, the DNA composition of *P. lagopus* genome with and without B chromosomes was in silico-characterized using advanced sequencing technology. With highly and moderately repeated elements, the nuclear genome (2.46 pg/2C) was found to be relatively rich in repetitive sequences, making up 68% of the genome [35]. A centromere-specific marker, a B-specific satellite and repeat enriched in polymorphic A chromosome segments were identified. The B-specific tandem repeat PLsatB was found to originate from sequence amplification including 5S rDNA fragments. The repetitive sequences found were classified into established groups of repetitive elements. Maximus/SIRE lineage of Ty1/Copia LTR-retrotransposons were reported to be the dominant repeat type, representing more than 25% of the genome (Figure 1).

## 5. Transcriptionally Active Sequences on B Chromosomes

It has been shown by various workers that B chromosomes are enriched with several classes of repetitive DNA elements [40,41]. Therefore, the identification of genes encoding proteins or the ones actively transcribing is tricky. However, the development of new techniques in recent years has considerably aided in identifying the protein encoding genes, pseudogenes, rRNA genes, and actively transcribing sequences on B chromosomes.

Based on the relationship of nucleolar phenotypes with rRNA gene expression, the first indirect evidence of active sequences on Bs was provided by Bidau [42]. However, Leach et al., provided the molecular evidence of gene activity on B chromosomes in *C. capillaris* [43]. Transcription of rRNA sequences located on B chromosomes was recently demonstrated [44,45]. Some of the important findings provided evidence that various repetitive DNAs are transcriptionally modulated by B chromosomes [37,46,47,48,49,50].

The proto-oncogene *c-KIT* has been mapped to B chromosomes of some animal species [51,52] and also in Siberian deer [53], where this gene is transcriptionally active. Studies on B chromosomes of Cichlid fishes have revealed many interesting facts. In *A. latifasciata*, Valente et al., have identified several genes on B chromosomes [6]. These include the genes that are involved in the chromosomal segregation and the proteins that are involved in i) microtubule organization, namely *TUBB1*, *TUBB5*; ii) kinetochore structure, such as *SKA1*, *KIF11*, and *CENP-E*, iii) recombination, including *XRCC2*, *SYCP2*, and *RTEL1*, and iv) progression through the cell cycle, such as *Separase* and *AURK* [6].

Although B chromosomes are known to affect the pairing behavior of As, recent studies have demonstrated that the presence of B chromosomes also influences the transcription of genes located on A chromosomes [47,49,54]. Huang et al., used RNAseq technique to analyze the expression of genes in maize plants with a variable number of B chromosomes and observed that the effect of the high number of B chromosomes is more pronounced [4]. Similarly, B chromosome of *A. latifasciata* regulates the expression of noncoding RNAs, thus influencing A genome expression [50].

Interestingly, in vitro activity of a B chromosome encoded protein gene that is possibly involved in regulation has been reported in rye, thereby providing strong evidence that a B derived protein could function in silencing chromatin/DNA elements [55].

Certain phenotypic effects have been ascribed to the presence of B chromosomes. When present in low numbers, Bs have little effect on the phenotype, but at higher numbers they mostly have a negative effect on fitness and fertility of the organism [3,56]. In general, Bs negatively affect the fitness and fertility of the plants [57]. Bs directly or indirectly influence the behavior of A chromosomes. An increase in pigment production in achene’s walls was observed in individuals carrying Bs in *Haplopappus gracilis* [58]. In maize, leaf stripping was correlated with the presence of Bs [59]. In *Allium schoenoprasum*, seeds carrying Bs have an advantage concerning germination over 0B seeds under drought stress conditions [60]. Bs also play a role in sex determination in cichlid fishes and in the frog *Leiopelma hochstetteri* [61]. In *P. coronopus*, the presence of Bs caused male sterility [62].

In grasshopper *Myrmeleotettix maculatus*, the presence of Bs was correlated with retardation in animal development [63] and sperm dysfunction [64]. Bs also lead to meiotic pairing in *Aegilops mutica* [65] and in hybrids between common wheat and *Aegilops variabilis* [66]. The presence of B has also been linked with crown rust resistance in *Avena sativa* [67]. In *Nasonia vitripennis*, the effect of B-like PSR chromosome constitutes changing the males into females by destroying the paternal chromosome during early embryogenesis [68]. In rice, Cheng et al., observed slight positive effect of Bs on plant height, length of panicle, length and weight of grain, and negative effect on number of tillers and width of grain [69]. In some species, individuals with Bs show better survival rate under certain stress conditions. In *A. schoenoprasum* [60], when grown under high sowing density, +B plants showed better fitness than 0B plants. A positive correlation between the mean number of B chromosomes and body mass has been observed in males of *Apodemus flavicollis* [70]. In hybrids, Bs prevent or suppress the homologous pairing of A chromosomes. This effect was observed in hybrids between *A. mutica* and *A. speltoides* with *Triticum aestivum* [65] and in a tetraploid hybrid between *Lolium temulentum* × *L. perenne* [71].

Using NGS-based approaches, it has been unfolded that Bs contain a high number of genic sequences. More than 4000 putative B-located genic sequences have been identified on rye B chromosome [34], which showed sequence polymorphism as compared to their A-located counterparts confirming pseudogenization [49]. In *D. albomicans*, one actively transcribed unit on B has been detected [38]. The evolutionary fate of these B-located genic sequences has been well addressed in a review by Houben et al. [1]. These authors have suggested that the dispensable nature of B chromosomes helps them to accumulate mutations, while they go through pseudogenization. Interestingly, all the B located genes are not inactive and also most of Bs have identical genic sequences with As, yet they still do not lead to severe phenotype. Moreover, the dosage of a chromosome is critical for normal development, but due to dosage compensation A-derived genes are likely to be downregulated [1].

## 6. Drive Mechanisms and Transmission of B Chromosomes

Various mechanisms by which B chromosomes are able to ensure preferential transmission to the progeny are known as accumulation or drive mechanisms. When the transmission rates of chromosomes are higher than 0.5 through male or female sex tracks, and the Mendel’s law is not followed, the resulting transmission advantage is jointly referred to as drive [5]. The molecular mechanisms that are responsible for the drive are unknown [41], although these are fundamental in understanding B chromosomes. Bs successfully accumulate in populations because their transmission frequency is higher than expected, though they are mostly devoid of essential genes. There are several other genetic elements reported, which, at the expense of other components of the genome, promote their own transmission e.g., spore killer in fungi, the t haplotype in mouse and segregation distorter (SD) in *Drosophila* [72]. Drive leads to an increased number of Bs in next generation and the maximal number of Bs that can be tolerated by the host varies between species e.g., maize has 34 while rye has six B chromosomes. In this regard, *P. lagopus* is unique in having 2 B chromosomes at diploid and 5 B chromosomes at triploid levels [73]. It depends on a balance between B chromosome accumulation due to drive, and negative effects on fertility and vigor that are caused by the B chromosome [5]. However, not all B carrying species possess a drive mechanism.

The drive can occur at any stage of life cycle and has accordingly been classified as pre-meiotic, meiotic, and post-meiotic. In pre-meiotic drive, B chromosomes increase in number in the germ line cells and the mean number of B chromosomes increases, when the latter enter meiosis to form gametes. In animals, the drive occurs either before or during meiosis as the gametic nuclei are not replicating. Premeiotic drive occurs in the spermatogonial mitosis in the testes in animals [74]. It has been observed that mitotic nondisjunction occurs and the cells that possess B chromosome are preferentially included in the germ line cells of grasshopper *Locusta migratoria*. In plants, premeiotic drive has been described in *C. capillaris*, with a difference that during development the mitotic nondisjunction occurs in the meristematic cells during flower initiation with the preferential inclusion of Bs in inflorescence [75].

Meiotic drive depends on the functional symmetry of meiotic products. There are reports on the existence of meiotic drive in some grasshopper species [76]. Meiotic drive has been seen in the megaspore mother cells of *Lilium callosum* [13], a species with tetrasporic embryo sac development. Here, Bs were observed on the micropylar side of the spindle in 80% of the analyzed cells due to which they were incorporated into the resulting egg cell. Similarly, in grasshopper *M. maculatus*, Bs were located at the egg pole rather than polar bodies pole [5].

Post-meiotic drive occurs immediately after meiosis during the development of the male and female gametophyte [75]. The molecular mechanisms that are involved in drive were not known for long, until recently when Banaei-Moghaddam et al., showed meiotic drive to be due to the non-disjunction of chromatids of the B chromosome [77]. Post-meiotic drive has been frequently observed in flowering plants during the male gametophyte maturation. In rye, nondisjunction occurs during the first pollen grain mitosis, which results in the accumulation of Bs, due to which higher number of Bs are transmitted to the next generation [5]. The generative nucleus divides to produce two sperm nuclei each with unreduced number of Bs during the second pollen grain mitosis. The process of nondisjunction and frequency of transmission is controlled by B itself [5]. While analyzing the B chromosome variants, a region controlling the process of non-disjunction at the end of the long chromosome was identified, which led to the accumulation of B chromosomes. The absence of non-disjunction control region (NCR) leads to normal disjunction [78]. This factor has been shown to act in trans in rye, because when a standard B was present in the same cell that contains B lacking terminal region, the standard B mediates not only its own nondisjunction, but also of the deficient B [78]. Several B-specific satellite DNAs are present in the heterochromatic NCR. The NCR has also been labelled with H3K4me3, which is a euchromatin-specific post-translational histone mark [5].

There has been no report of a gene or a DNA sequence characterized that may be responsible for the accumulation of B chromosome. Recently, noncoding RNA has been surmised to be responsible for nondisjunction [1] because of its role in fission yeast [79] and flies [80]. It has been observed that long-non-coding RNA is produced by some NCR specific satellites predominantly in anthers [46]. There is a possibility that B chromosome encoded non-coding RNAs block necessary factors at specific genomic loci e.g., the B pericentromere or the B-derived noncoding RNAs could act as guide molecules that direct protein complexes [77]. The recent reports on identification of a notably high number of B-encoded transcripts in a number of species, furnish the basis to hypothesize the involvement of protein-coding genes, or pseudogenes for controlling non-disjunction e.g., in rye [34,54], fish [50], *Drosophila* [81], and cervids [82].

The mechanism of accumulation of the B chromosome in *P. lagopus* was recently reported in depth by Dhar et al., (2017) by performing extensive crossing experiments and calculating the transmission of B chromosome through female and male sex tracks [73]. This study not only explored the existence of drive in B chromosome of *P. lagopus*, but also helped in understanding the mechanism of perpetuation in the populations. After selfing and crossing, the progenies were raised and analyzed to determine the mode of transmission of B chromosomes. Crosses were attempted between the 0B, 1B, and 2B plants. About 531 plants were screened cytologically for the presence of the B chromosome; FISH with 5S rDNA probe was used to identify the B chromosome(s) in 1B and 2B plants.

It was reported that in *P. lagopus* when 1B plant was used as a male, transmission rate was in accordance with the expected Mendelian ratio. The differences in segregation ratio observed among various cross combinations were attributed to the heterozygous nature of *P. lagopus*—being a cross-pollinated species [73]. Alternatively, when 1B plants were used as female, there were significant deviations from the 1:1 ratio; frequency of B chromosome bearing plants was higher than the Mendelian expected value. These results indicated the preferential transmission of B chromosome through the female sex track in *P. lagopus*. Such variation in the transmission rate is a common feature of B inheritance, such that the Bs tend to distort Mendelian expectation in their favor [83]. The fact that during female meiosis in plants, only one out of the four meiotic products survives can explain this drive through the female sex tracks. The B chromosome of *P. lagopus* is perpetuated preferentially due to drive, expressed during both male and female meiosis [73]. The same phenomenon has been observed in rye where the drive happens in both male and female sex tracks [84].

In *P. lagopus*, the entire complement of the species gets duplicated in the presence of a B chromosome [73]. It was for the first time that such a phenomenon has been reported in plants, earlier, the presence of macrospermatids (> diploid chromosome number) in B containing individuals of a grasshopper *Dichroplus pratensis* were observed [85]. The formation of macrospermatids was attributed to nuclear fusion. In *P. lagopus* (2n = 12), plants with 23, 26, and 28 chromosomes were obtained from the selfed progenies of B chromosome bearing plants. The origin of these was attributed to the formation of unreduced gametes, followed by their fusion with other gametes or their endoreduplication and parthenogenetic development of the plant.

## 7. B Chromosome in Relation to Breeding System

The breeding system plays a fundamental role in the ecology and evolution of B chromosomes. In a comparative study performed by Burt and Trivers among different species of British flowering plants, it was demonstrated that Bs are more likely to be present in outbreeding species than in inbreeding species [86]. Besides, there are a number of other factors that play an important role in the prevalence of B chromosomes, like genome size, chromosome number, and ploidy level [22]. In several cases, experiments have verified the relationship between Bs and outbreeding. For example, when outbreeding rye (*Secale cereale*) was inbred, the B frequency was observed to decline [87]. Similarly, in inbreeding *Secale vavilovii*, when B chromosomes were experimentally introduced, their number declined rapidly [88]. Since the variation in the number of Bs among individuals gets increased in inbreeding, the latter in turn decreases B frequency by increasing the power of natural selection. Genus *Plantago* has a wide range of mating systems, from inbreeders to obligate outcrossers. Outbreeding species, such as, *P. lanceolata*, *P. lagopus*, *P. coronopus*, and *P. depressa* possess B chromosome. On the contrary, *P. ovata* and *Plantago patagonica*, representing inbreeding types are devoid of B chromosome—a fact that further substantiates the relation between the breeding system and B chromosome existence.

In order to understand the relationship between the breeding system and B chromosome, reference can be made to the data given by Levin et al., wherein phyletic hotspots for B chromosomes in Angiosperms has been discussed in detail [17]. Further, the prevalence of B chromosomes across different orders in Angiosperms is noteworthy. From there, it can be inferred that Asparagales, Liliales and Poales (Monocots), Ranunculales (Eudicot), Fabales (Rosids), and Asterales (Euasterids) had maximum number of B chromosomes. All of the species belonging to these orders having maximum Bs are outbreeders [86].

Several species of *Plantago*, including *P. lanceolata* and *P. lagopus*, which possess the B chromosome, follow outcrossing and are also gynodioecious [89]. The role of gynodioecy as an outcrossing mechanism was well demonstrated while understanding the gynodioecious breeding system in *P. lanceolata* [90], where it was observed that females produce 1.77 times the number of seeds produced by hermaphrodites over the flowering season. The superior reproductive output of females in gynodioecious population is required for the maintenance of gynodioecy [91]. However, there must be some selective advantage of the B possessing plants, which helps them in attaining and maintaining gynodioecy. It can be conjectured that organellar genes that have been reported to be present on B chromosome confer this advantage. Nuclear transfer of organelle DNA is a known process. There is a frequent transfer of mitochondrial and chloroplast DNA sequences into the nuclear genome [92]. Nuclear insertions of plastid DNA (NUPTs) and insertions of mitochondrial DNA (NUMTs) have been shown to be involved in the formation of new nuclear genes. There are a number of examples where organellar sequences have been reported on B chromosome. Large insertions of organelle DNA were found on B chromosomes of rye, *A. speltoides* [93] and wheat [34]. Recently, using techniques like next-generation sequencing and sophisticated bioinformatics tools, it was observed that in rye [34] and in the fish species *A. latifasciata* [6], B chromosomes have accumulated organellar-derived DNA [35]. There is a possibility that these captured organellar sequences have a role in affecting the sex phenotypes of gynodioecious systems, and manipulate their transmission advantage.

## 8. Evolution of B Chromosomes

Despite widespread occurrence of B chromosomes in all eukaryotic groups, including plants, mammals, insects, etc., the origin of B chromosome has remained an enigma. Several hypotheses have been proposed for the origin of B chromosomes [12,74,84,94]. Bs seem to have arisen in different ways in different organisms [1,74,84]. The view that is most widely accepted is that they are derived from A chromosome complement. There are some evidences that suggest that B chromosomes were generated spontaneously in response to the new genomic conditions after interspecific hybridization [74]. In some animals, the involvement of sex chromosomes has also been argued for their origin [41,74,84]. Moreover, the presence of similar B chromosome variants within related species suggests that the B chromosome arose from a single origin [34]. It has been argued that B chromosomes are selfish entities that take a distinct path of evolution and differ in sequence composition from that of the As [1]. The mobile elements and other repeats on B chromosome can easily spread and amplify, because B chromosomes are not under selective pressure. This has been reported in Bs of *B. dichromosomatica* [32], *P. lagopus* [10,12], and *Zea mays* [37].

In a historical paper from our laboratory [12], the first experimental proof of the origin of B chromosome from A chromosome in *P. lagopus* was provided. The model proposed was widely accepted and adapted [14,83]. Dhar et al., proposed that the B chromosome arose as a result of the massive amplification of sequences with similarities to 5S and 45S rDNA, as well as to other sequences [12]. The C–banding analysis clearly distinguished the B chromosome from other chromosomes in being entirely heterochromatic. Recently, NGS and Graph based clustering analysis for characterization of DNA composition of *P. lagopus* with and without B chromosomes was performed [35], that helped a great deal in ascertaining the validity of the model proposed by Dhar et al., [12]. Centromere-specific marker, a B-specific satellite, and a repeat enriched in polymorphic A chromosome segments have been identified.

In *P. lagopus*, A and B chromosomes differ significantly in composition, which is mainly due to an additional massive amplification of B-specific satellite repeats. The proportion of satellite repeats in 0B plants is 3.5% and in +B plants 6.7% [35]. The same sequences are also abundant in the A chromosomes, particularly in heterochromatic regions and it is these repetitive elements that contribute to the heterochromatic nature of the B chromosome. Cluster CL4, a satellite, with a monomer length of 325 bp, shared similarity with 5S rDNA. It had more proportion of reads from +B DNA i.e., 2.95% and very less proportion of 0B reads i.e., 0.08%. This indicates that the sequences present in this cluster were specific to B chromosome. When closely inspected, these sequences showed some similarity to 5S rDNA, but were not 5S sequences. This cluster can be fairly linked with the experimental evidence provided earlier by Dhar et al., where Southern blot analysis revealed a distinct band of about 23 kb, only in +B plants [12]. Since this cluster is specific to reads that are present on B chromosome as was the 23 kb fragment specific to +B DNA, a very important and relevant correlation can be drawn between the two.

Very early attempts to elucidate the DNA composition of Bs resulted in comparative studies of 0B versus +B genomic DNA [95,96,97,98]. However, during the last few years, microdissection [32] and flow sorting [34] have helped in the reliable isolation of B-derived DNA. B chromosomes of many species contain sequences that originated from one or more A chromosomes [32,35]. In the harvest mouse, *Reithrodontomys megalotis*, different origins for the two types of Bs found were suggested. The large B chromosome arose from centric fusion as a leftover of the centromere, whereas the small B had originated from an amplified region of an A chromosome or as an intact fragment. Karamysheva et al., proposed a two-step appearance of Bs in *Apodemus peninsulae* [99]. Destabilization of pericentromeric regions that are produced by the invasion of DNA sequences from euchromatic parts of A chromosomes leads to the formation of micro-chromosomes in high frequency, which could be considered as proto Bs. The next step is the insertion and amplification of new DNA sequences.

It is not clear how the actual process of sequence transfer from As to Bs takes place, but recent reports indicate that the transposition of mobile elements may be playing an important role [100]. B chromosomes are an ideal target for the transposition of mobile elements [48], and the structural variability that is observed in Bs can be due to the insertion of these elements [74]. In maize, when a large DNA insert clone was analyzed, Bs were observed to be composed of B-specific sequences that were interlaced with those in common with the As [37]. StarkB elements (22 kb-long B-specific repeat) had frequent insertions by LTR-type retroelement [37]. Within evolutionarily diverged species, the presence of B-enriched sequences indicated that B-specific amplification occurred after separation from the standard chromosome complement [92].

In rye that was collected from several countries, Bs have been reported in both cultivated and weedy forms [101]. In F1 hybrids of these forms, Bs had similar morphology and meiotic pairing which suggested that the rye B has a monophyletic origin [101]. The comparative sequence analysis of the A and B chromosomes in rye revealed that Bs are rich in gene fragments that are derived from multiple A chromosome fragments [102]. Many short sequences that are similar to other regions of the rye A supported the multi-chromosomal origin of B-chromosome sequences. When the composition and distribution of high-copy sequences were explored, it got affirmed that Bs contain a similar proportion of repeats as in the A chromosomes, but differ substantially in repeat composition [48]. The distribution of mobile elements, like Gypsy and Copia, in the genome of rye is similar along As and Bs [103]. Accumulation of Gypsy retrotransposons or other repeated sequences on B chromosome has also been reported in the fish *A. alburnus* [29] and the fungus *N. haematococca* [104].

In various organisms, it has been observed that Bs also contain various kinds of coding and noncoding repeats, which are very similar to those that are found in circular extrachromsomal DNA [105]. In *B. dichromosomatica*, extrachromosomal DNA with similarity to tandem repeat sequences shared by both A and B chromosomes have been identified [106]. However, whether an evolutionary link between extrachromosomal DNA and the evolution of Bs exists is still to be determined. In wild canid species, several regions of domestic dog sequences that share sequence similarity with canid B chromosomes were identified by analyzing evolutionarily conserved chromosome segments [102].

The involvement of rDNA in the evolution of B chromosomes does not appear to be accidental, as they have been detected on Bs of many plants (e.g., *C. capillaris* [107] as well as animals (e.g., *Rattus rattus* [108]). The most convincing example is of *P. lagopus* where the analysis of many generations and in situ localization with rDNA probes revealed that B originated as result of massive amplification of 5S rDNA [12,35]. It has been argued that the mobile nature of rDNA could be the reason for their presence as landing sites on the B chromosomes [1]. The reason for no or weak transcription of rDNA on B chromosomes is hazy, although differences in the histone H3 methylation between A and B chromosome may be responsible [109]. By determining the relatedness of internal transcribed spacers (ITS) between the different chromosome types, the available sequence information for B-located rRNA genes has been used to study the origin of Bs e.g., *B. dichromosomatica* [85] and *C. capillaris* [43]. According to Martis et al., Bs have been found to have accumulated large amounts of B-specific repeats and insertions of cytoplasmic organellar DNA in A-derived landscape [34]. They have termed Bs as genomic sponge, which collects and maintains sequences of diverse origin.

Some findings suggest that B chromosomes arise spontaneously in response to genomic stress following interspecific hybridization [110]. First proposed by Battaglia (1964), this mode of origin was later confirmed by other scientists [74,111]. In hybrids generated after interspecific crosses between *Coix aquaticus* (2n = 10) and *Coix gigantea* (2n = 20), a spectrum of individuals with variable number of *gigantea* and *aquaticus* chromosomes were recovered. *C. gigantea* chromosomes in *C. aquaticus* genome behaved like B chromosomes during meiosis [14]. It is presumed that an incomplete loss of one parental genome, during hybrid embryogenesis, might have played a role in the origin of Bs. There is evidence that the centromeres of the parental chromosomes undergoing elimination are the last to be lost during the uniparental chromosome elimination process [112]. A novel mechanism for the evolution of B chromosome based on the recombination of nonhomologous chromosomes, which takes place during the DNA double-strand repair process at S-phase, has been postulated for the formation of zebra chromosome, which is composed of *T. aestivum*/*Elymus trachycaulus*, structurally rearranged chromosome fragments [113]. In the gynogenetic fish *Poecilia formosa*, the sperm of male from the related bisexual species *Poecilia mexicana* or *Poecilia latipinna* are required for the initiation of embryogenesis. Eventually, paternal genome is eliminated early in the development. The progeny results in a spotted pigmentation phenotype. Supernumerary microchromosomes were observed after cytological analysis in *P. formosa* offsprings as a result of incomplete elimination of paternal genome. They are inherited and variable pigmentation results due to the different number of Bs [16].

B chromosomes may also be derived from sex chromosomes [38] during intraspecific origin. There are many features, like distribution of chromatin, the accumulation of repetitive DNA, and the loss of gene activity, which are shared by both sex and B chromosomes. The origin of Bs from sex chromosomes has also been suggested because of the similarity of morphology, heteropycnocity, and meiotic behavior between Bs and sex chromosomes in some species [74]. The similarity in the composition and localization of two DNA probes (180 bp tandem repeat and ribosomal DNA) of B chromosomes, which are mostly composed of these two sequences, with those on X chromosome suggest that the B chromosome in grasshopper *E. plorans* probably originated from X chromosomes [114]. The B chromosomes of *L. hochstetteri* showed similarity to the W sex chromosome at the DNA level [115]. Morphological similarities between Bs and the univalent W chromosome were also observed [116]. Genus *Characidium* among Neotropical fishes provides an intriguing model for cytogenetic and evolutionary studies, principally because of the presence of differentiated sex chromosome systems and B chromosomes [117]. DNA probes that were obtained from B chromosomes and sex chromosomes in three species of fish genus *Characidium* were used for chromosome painting, which showed a close resemblance in repetitive DNA content between B and sex chromosomes. Bs in Bandicoot (*Echymipera kalubu*) have been reported to be derived from sex chromosomes, based on the finding that Bs follow the same fate as sex chromosomes, which are eliminated from certain somatic tissues [15]. Frequent synapsis and recombination between Bs and the Y chromosome in *Dicrostonyx groenlandicus* advocate Y chromosome as a possible source of Bs [118].

## 9. Outlook

A number of efficient tools for the analysis of B chromosomes have been developed due to current breakthrough in gene sequencing and bioinformatics. Further analysis of composition and behavior of B chromosomes will help in tracing the origin of these enigmatic chromosomes. We would have a better discernment as to how selection pressure leads to genome evolution. In different organisms where the molecular studies on B chromosomes has been conducted, various theories have been propounded regarding the evolution of Bs. Having a thorough knowledge of Bs will give us exhilarating results about frequent changes in higher eukaryotes, with special reference to these supernumerary chromosomes.
